# Vacuum-Assisted Incisional Closure Therapy After Groin Reconstruction With Muscle Flap

**DOI:** 10.7759/cureus.14954

**Published:** 2021-05-11

**Authors:** Maryclare E Taylor, Ledibabari M Ngaage, Philip Wasicek, Michael Ha, Khanjan Nagarsheth, Shahab A Toursavadkohi, John Karwowski, Yvonne M Rasko

**Affiliations:** 1 Plastic and Reconstructive Surgery, University of Maryland School of Medicine, Baltimore, USA; 2 General Surgery, University of Maryland School of Medicine, Baltimore, USA; 3 Vascular Surgery, University of Maryland School of Medicine, Baltimore, USA

**Keywords:** wound closure, wound repair, groin, surgical flaps, negative pressure wound therapy, reconstructive surgical procedures

## Abstract

Background

Groin reconstruction with muscle flap coverage is associated with high wound complication rates. Incisional vacuum-assisted closure (iVAC) therapy may lower wound complications. We evaluated the impact of iVAC on postoperative outcomes in patients following groin reconstruction with muscle flap coverage.

Methods

We conducted a retrospective review of patients who underwent groin reconstruction with muscle flap coverage in 2012-2018. Patients were divided into those who received iVAC therapy and those who received standard sterile dressings (SSD).

Results

Of the 57 patients included, most received iVAC therapy (71%, *n *= 41) and the rest received SSD (28%, *n *= 16). The iVAC group had higher rates of diabetes, hypertension, coronary artery disease, and peripheral artery disease (*p *< 0.05). However, iVAC patients had comparable length of hospital stay (12 vs 8.5 days *p = *0.0735), reoperations (34% vs 31%, *p *= 0.8415), and readmissions (32% vs 37%, *p *= 0.6801) with SSD patients. iVAC placement was less likely in prophylactic flaps (odds ratio 0.08, *p *= 0.0049).

Conclusion

Patients with a prophylactic flap were less likely to receive vacuum therapy, which may highlight a selection bias where surgeons pre-emptively use iVAC therapy in surgical candidates identified as high risk. The pre-emptive use of iVAC may minimize adverse postoperative outcomes in high-risk patients.

## Introduction

Wound complications after major surgery on the groin are a serious problem associated with significant morbidity and cost. Complication rates as high as 44% have been reported in infra-inguinal vascular procedures [1-3]. Surgery on the groin puts a patient at particular risk of infection, wound dehiscence, lymphatic leaks, and hematomas [2,4]. Vascular patients with the presence of comorbidities such as obesity, cigarette use, diabetes, and prior groin surgery are at increased risk of complications [1]. In addition to causing pain and distress to the patient, complications are a significant source of healthcare costs. Surgical site infections (SSIs) incur on average 3.8 additional days in the hospital [5]. Furthermore, the cost of hospitalization in a patient with an SSI was estimated to be $8,909 higher than the cost for a patient without an SSI [5-6].

Several methods have been proposed to reduce the complication rates in groin surgery. The use of muscle flaps to treat complications following vascular surgery on the groin was first described in 1989 by Mahoney et al. and has become a widely accepted method of salvage therapy [7-13]. More recently, early use of prophylactic muscle flaps in high-risk patients has been extensively studied and demonstrated to have favorable outcomes [1,13]. However, even with prophylactic utilization of muscle flap coverage in high-risk patients, complication rates of 16% have been reported [1].

Another approach to complication rate reduction in groin surgery is with the use of vacuum-assisted incisional closure therapy (iVAC). The use of iVAC therapy is thought to provide benefits and decrease complication rates by acting as a barrier to the incision from external infectious sources, helping promote skin edge apposition, remove fluid and infectious materials from the incision, and increase microcirculation [2,14-18]. A recent meta-analysis of iVAC therapy in patients undergoing groin vascular surgery has shown a statistically significant improvement in outcomes over traditional dressings [6]. Yet, there is no literature examining the use of iVAC therapy on patients with prophylactic or salvage muscle flap groin reconstruction.

The purpose of this study was to delineate the utility of the regular use of iVACs in groin reconstruction via retrospective analysis of patients who have undergone groin reconstruction with muscle flap coverage.

## Materials and methods

**Figure 1:**
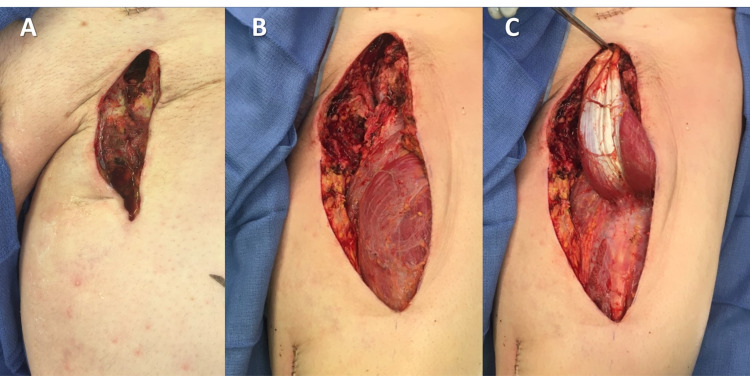
Example groin reconstruction via rectus femoris flap (A) Initial vascular necrotic wound. (B) Exposed bypass graft. (C) Elevation of rectus femoris flap.

This project was approved by the University of Maryland Institutional Review Board. We performed a retrospective review of all patients who had received groin reconstruction from December 2012 to December 2018. Eligible patients were identified with current procedural terminology (CPT) codes for muscle flap coverage and groin reconstruction (CPT15738 and CPT49568, respectively). An example case is presented in Figure 1. Patients who did not have groin reconstruction but only muscle flap advancement or bilateral abdominal muscle release for ventral hernias were excluded (n=16).

**Figure 2:**
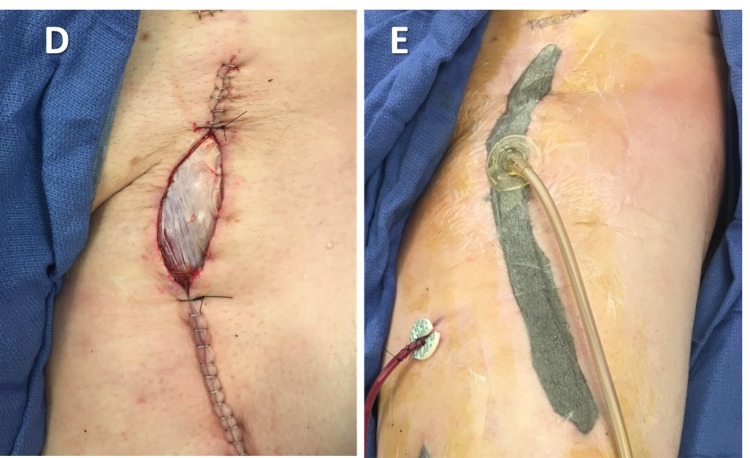
Closure of a rectus femoris flap (D) Partial closure via staples. (E) Overlaying vacuum-assisted incisional closure therapy (iVAC) dressing.

Patients were divided into two groups: vacuum-assisted incisional closure (iVAC) (Figure 2) and standard sterile dressing (SSD), which consisted of a dry gauze dressing. We collected details on patient demographics, operative detail, and postoperative course. The primary outcome was postoperative surgical site complications. Surgical site complications included surgical site infections, wound dehiscence, skin necrosis, non-healing incisional wound, seroma, and hematoma. Complications were graded according to the Clavien-Dindo classification [19]. Operative intervention was defined as any surgical site complication that required the opening of the wound, wound debridement, or percutaneous drainage. Secondary outcomes were the length of hospital stay and unplanned readmissions within 90 days.

Composite data were stored in Microsoft Excel (Microsoft Corporation, Redmond, Washington) and analyzed using the Statistical Package for the Social Sciences (SPSS) software version 26 (IBM Corp, 2019. Armonk, NY). Non-parametric variables were summarized and analyzed using median values and interquartile ranges. Categorical data were summarized using percentages. Differences in continuous data between the groups were tested with the non-parametric Mann-Whitney-U test. The chi-squared test and Fisher exact test were used to test for differences between categorical data, as appropriate. The odds ratio (OR) is reported with 95% confidence intervals (95% CI). Variables found to be significantly different between the two groups in univariate analysis at p<0.25 and clinically relevant variables as determined by clinical judgment were included in the multinomial logistic regression. Variables were checked for multicollinearity (variance inflation factor, VIF) and were excluded if multicollinearity was found (VIF >1.5). Statistical significance was defined as a two-tailed value of p≤0.05.

## Results

Fifty-seven patients were included in our study. Nearly three quarters received an iVAC (71%, n=41) and the rest received an SSD (28%, n=16). The patient and surgical characteristics of the two study groups are summarized in Table 1. The two study groups were similar in terms of age, gender, body mass index (BMI), and race. However, significant differences in patient comorbidities were observed between the two groups. Patients in the iVAC group had higher rates of diabetes (46% vs 13%, p=0.0174), hypertension (61% vs 31%, p=0.0434 ), coronary artery disease (39% vs 6%, p=0.0221), and peripheral artery disease (46% vs 0%, p=0.0011) than patients in the SSD group.

**Table 1:** Patient demographics CAD, coronary artery disease; PAD, peripheral artery disease; HTN, hypertension; IBD, inflammatory bowel disease; BMI, body mass index; SSD, standard sterile dressing; iVAC, vacuum-assisted incisional closure device; SD, standard deviation; Q1; Q3, first quartile, third quartile; recon, reconstruction

Patient Demographics				
Variable	Total (n=57)	iVAC (n=41)	SSD (n=16)	p-value
Mean Age / years (SD)	53.8 (17.7)	56.2 (18.6)	47.8 (13.8)	0.0674
Gender				0.3801
Male	34 (60%)	26 (63%)	8 (50%)	
Female	23 (40%)	15 (37%)	8 (50%)	
Mean BMI (SD)	27.4 (7.2)	27.7 (7.8)	26.6 (5.4)	0.5976
Race				0.5408
Caucasian	37 (64%)	28 (69%)	9 (56%)	
African American	19 (33%)	12 (29%)	7 (44%)	
Other	1 (1%)	1 (2%)	0 (0%)	
Medical history				
Diabetes	21 (37%)	19 (46%)	2 (13%)	0.0174
CAD	17 (30%)	16 (39%)	1 (6%)	0.0221
PAD	19 (33%)	19 (46%)	0 (0%)	0.0011
HTN	30 (53%)	25 (61%)	5 (31%)	0.0434
Cigarette use	45 (79%)	35 (85%)	10 (63%)	0.0762
Previous groin surgery	44 (77%)	35 (85%)	9 (56%)	0.0327
Timing of flap				
Salvage	35 (60%)	32 (78%)	3 (19%)	<0.0001
Prophylactic	23 (40%)	9 (21%)	13 (81%)	
Operative indication				-
Infection	27 (47%)	26 (63%)	1 (6%)	
Hemorrhage	1 (2%)	1 (2%)	0 (0%)	
Oncologic recon	11 (19%)	2 (5%)	9 (56%)	
Orthopedic recon	5 (9%)	5 (12%)	0 (0%)	
Trauma recon	2 (4%)	1 (2%)	1 (6%)	
Vascular recon	7 (12%)	6 (15%)	1 (6%)	
IBD recon	4 (7%)	0 (0%)	4 (25%)	
Flap type				-
Gracilis	15 (26%)	2 (4%)	13 (81%)	
Rectus femoris	14 (24%)	13 (31%)	1 (6%)	
Sartorius	17 (30%)	17 (41%)	0 (0%)	
Tensor fascia lata	1 (2%)	1 (2%)	0 (0%)	
Fasciocutaneous	1 (2%)	0 (0%)	1 (6%)	
Gastrocnemous	1 (2%)	1 (2%)	1 (6%)	
Soleus	1 (2%)	1 (2%)	0 (0%)	
Vastus lateralis	7 (12%)	6 (15%)	1 (6%)	
Vastus medialis	1 (2%)	1 (2%)	0 (0%)	
Other	4 (7%)	3 (7%)	1 (6%)	
Median follow-up time / days (Q1, Q3)	547 (67, 836.5)	399 (44, 819.5)	679 (91, 919.5)	0.0093
Mean follow-up time / days (SD)	513.7 (453.1)	402.5 (387.5)	798.6 (488.1)	0.0023

A greater proportion of patients in the iVAC group had a history of prior groin surgery as compared to the SSD group (85% vs 56%, p=0.0327). Additionally, 78% of patients who underwent iVAC placement had salvage, rather than prophylactic, muscle flaps as compared to 19% of patients in the SSD group with salvage muscle flaps (p<0.0001). There were increased odds of iVAC use with salvage flaps (OR=15.41, 95% CI: 3.59-66.15, p=0.0002). Groin reconstruction for infective indication was more common in patients who underwent iVAC placement than the SSD group (63% vs 6%, p<0.0001), whereas oncologic reconstruction was significantly more prevalent in the SSD group (56% vs 5%, p<0.0001). The most common muscle flaps utilized in the iVAC group were the sartorius (29%, n=17), gracilis (28%, n=16), and rectus femoris (31%, n=13), whereas the SSD group almost exclusively received a gracilis muscle flap (81%, n=13).

The comparison of postoperative outcomes is shown in Table 2. The mean operative time for patients with iVAC placement was significantly less than for patients receiving SSD (199.6 minutes vs 364 minutes, p<0.0001). There was no significant difference in the number of patients who received postoperative blood transfusions between the iVAC and SSD groups (20% vs 19%, p=1.0000). Patients with iVAC placement had comparable median hospital stays to those in the SSD group (12 vs 8.5, p=0.0735). There was also no significant difference between the iVAC and SSD rates of surgical site complications (39% vs 31%, p=0.5838). Similarly, the iVAC and SSD groups had similar rates of operative interventions to treat complications (34% vs 31%, p=0.8415) and readmissions within 90 days (32% vs 37%, p=0.6801).

**Table 2:** Perioperative characteristics SSD, standard sterile dressing; iVAC, vacuum-assisted incisional closure device; SD, standard deviation; Q1; Q3, first quartile, third quartile

Perioperative characteristics				
Variable	Total (n=57)	iVAC (n=41)	SSD (n=16)	p-value
Mean operative time / minutes (SD)	246.6 (147.5)	199.6 (115.7)	364 (154.9)	<0.0001
Patients who received blood transfusion	11 (16%)	8 (20%)	3 (19%)	1.0000
Median length of hospital stay / days (Q1, Q3)	11 (7, 20)	12 (8, 21)	8.5 (5.75, 12)	0.0735
Surgical site complications	21 (37%)	16 (39%)	5 (31%)	0.5838
Infection	7 (12%)	3 (7%)	4 (25%)	
Wound dehiscence	4 (7%)	2 (5%)	2 (13%)	
Skin necrosis	3 (5%)	2 (5%)	1 (6%)	
Chronic wound	3 (5%)	2 (5%)	1 (6%)	
Seroma	2 (4%)	2 (5%)	0 (0%)	
Hematoma	3 (5%)	3 (7%)	0 (0%)	
Other	2 (%)	2 (5%)	0 (0%)	
90-day operative intervention	19 (33%)	14 (34%)	5 (31%)	0.8415
Readmissions	19 (33%)	13 (32%)	6 (37%)	0.6801

Based on univariate analysis and clinical judgment, we identified variables of interest for multinomial logistic regression. Infection as an operative indication was found to be collinear with salvage flaps (VIF = 2.27) so was excluded from the analysis. We assessed risk factors for surgical site complications, reoperations, and readmissions (Table 3). The use of iVAC therapy did not significantly change rates of complications (OR=0.86, 95% CI: 0.16-4.62, p=0.8630), 90-day reoperations (OR=0.52, 95% CI: 0.08-3.52, p=0.5044), or 90-day readmissions (OR=0.85, 95% CI: 0.15-5.01, p=0.8581). However, diabetes was shown to be a risk factor for surgical site complications (OR=5.52, 95% CI: 1.13-27.17, p=0.0356) and 90-day reoperations (OR=18.1, 95% CI: 2.38-137.25, p=0.0051). Patients with a previous groin surgery had lower odds of experiencing a complication that required operative intervention (OR=0.10, 95% CI: 0.01-0.94, p=0.0437).

**Table 3:** Multivariate analysis for risk factors for surgical site complications CAD, coronary artery disease; CI, confidence interval; HTN, hypertension; iVAC, incisional vacuum-assisted closure therapy; OR, odds ratio; PAD, peripheral artery disease

Multivariate analysis for risk factors for surgical site complications						
Variable	Surgical site complication		90-day reoperations		90-day readmissions	
	OR (95% CI)	p-value	OR (95% CI)	p-value	OR (95% CI)	p-value
iVAC	0.86 (0.16 – 4.62)	0.8630	0.52 (0.08 – 3.52)	0.5044	0.85 (0.15 – 5.01)	0.8581
Medical history						
Diabetes	5.52 (1.13 – 27.17)	0.0356	18.1 (2.38 – 137.25)	0.0051	5.11 (0.86 – 30.46)	0.0735
CAD	0.66 (0.11 – 4.13)	0.6552	0.34 (0.04 – 2.68)	0.3033	2.91 (0.39 – 21.62)	0.2977
PAD	0.63 (0.08 – 4.94)	0.6652	0.85 (0.08 – 9.24)	0.8928	0.45 (0.05 – 3.90)	0.4685
HTN	1.77 (0.33 – 9.52)	0.5081	0.91 (0.12 – 7.19)	0.9302	0.79 (0.14 – 4.52)	0.7904
Smoking	6.42 (0.77 – 53.82)	0.0863	7.67 (0.75 – 78.53)	0.0860	5.72 (0.68 – 47.79)	0.1074
Previous groin surgery	0.42 (0.07 – 2.64)	0.3560	0.10 (0.01 – 0.94)	0.0437	0.95 (0.16 – 5.68)	0.9590
Salvage flap	1.00 (0.18 – 5.71)	1.0000	2.43 (0.29 – 20.03)	0.4096	0.23 (0.04 – 1.50)	0.1241

## Discussion

The aim of this study was to evaluate the utility of iVAC use in patients with muscle flap reconstruction of the groin. Our results show that iVAC therapy in groin reconstruction with muscle flap has comparable outcomes to standard sterile dressings despite use in a higher risk patient population. Furthermore, diabetes mellitus is the most important predictor for postoperative complications and reoperations within this patient population.

In our patient population, patients who received iVAC therapy were more likely to have risk factors such as diabetes, hypertension, coronary artery disease, and peripheral artery disease. Our results demonstrate that diabetes was an independent risk factor for surgical site complications and 90-day reoperations, which is consistent with previous literature [1,20-21]. Additionally, iVAC use was more common in patients receiving free flaps for infective indications. These patients are often critically ill, require long-term antibiotics, and have been found to have mortality rates ranging from 6% to 75 % [22]. The decision to utilize iVAC therapy was surgeon-dependent. iVAC therapy is thought to improve wound healing by creating a protective barrier, removing fluid and infectious materials from the incision, providing a better approximation of wound edges with decreased lateral tissue tension, and a potential increase in micro-circulation and oxygen saturation [2,14-18]. Furthermore, previous studies have found iVAC use to be associated with lower rates of surgical site infections than traditional dressings in high-risk patients [2,20,23]. Knowledge of this data may have led to a surgeon selection bias in which iVAC therapy was preferentially used in patients considered higher risk.

Additionally, patients were more likely to receive iVAC therapy in salvage groin reconstruction surgery, rather than prophylactically. The use of muscle flap as salvage therapy in wound complications of the groin has a well-studied benefit due to increased vascularization of the complication site, elimination of wound dead space, and increased lymphatic drainage around the site of transposition [1,7-10,12-13]. More recently, studies have proposed that the use of prophylactic muscle flaps may decrease complication rates in groin reconstruction [1]. The increased use of iVAC therapy in patients undergoing salvage muscle flap procedures may be secondary to surgeon bias. Our data reflects this bias, as the patients who underwent iVAC therapy were more likely to have groin flap reconstruction due to infectious indications.

Multivariate analysis demonstrated that iVAC did not influence surgical site complications, 90-day reoperations, or 90-day readmissions. This calls into question whether iVAC, which can cost up to $495, conveys a significant enough benefit to outweigh the inexpensive standard sterile dressing [2]. Further studies with larger patient populations and randomization of therapy as well as cost-benefit analyses are necessary to elicit the impact of iVAC therapy on groin complications and the economic utility of this therapy.

There are several limitations to our study. A major limitation is the introduction of selection bias, as patients were not randomized to iVAC vs SSD and the use of iVAC-assisted therapy was based on the surgeon’s discretion. Patients who were at higher preoperative risk were more likely to receive iVAC therapy. This is evidenced by the higher rates of diabetes, coronary artery disease, peripheral artery disease, and hypertension in the iVAC group. These factors may account for comparable outcomes between the iVAC and SSD groups. Patients with a prophylactic flap were less likely to receive iVAC therapy, which may further highlight selection bias in which surgeons pre-emptively use iVAC therapy in patients with pre-existing wound complications. This study also included patients treated by several different surgeons in varying surgical specialties, including general surgery, plastic surgery, and vascular surgery. Thus, inter-operator variability may be a confounding factor that was not accounted for in our analysis.

## Conclusions

Patients who received iVAC therapy had comparable complication, reoperation, and readmission rates to those who received standard dressings despite the possible higher preoperative risk for complications amongst the iVAC therapy group. The use of vacuum-assisted incisional closure therapy may be linked to a selection bias by the surgeon and used preferentially in patients perceived to be at high risk for complications after muscle flap groin reconstruction. The pre-emptive use of iVAC may minimize adverse postoperative outcomes in high-risk patients.
